# Foveal Therapy in Blue Cone Monochromacy: Predictions of Visual Potential From Artificial Intelligence

**DOI:** 10.3389/fnins.2020.00800

**Published:** 2020-08-03

**Authors:** Alexander Sumaroka, Artur V. Cideciyan, Rebecca Sheplock, Vivian Wu, Susanne Kohl, Bernd Wissinger, Samuel G. Jacobson

**Affiliations:** ^1^Scheie Eye Institute, Department of Ophthalmology, Perelman School of Medicine, University of Pennsylvania, Philadelphia, PA, United States; ^2^Molecular Genetics Laboratory, Institute for Ophthalmic Research, Centre for Ophthalmology, University of Tüebingen, Tüebingen, Germany

**Keywords:** machine learning, random forest, optical coherence tomography, chromatic perimetry, retinal degeneration, rods, cones, visual acuity

## Abstract

Novel therapeutic approaches for treating inherited retinal degenerations (IRDs) prompt a need to understand which patients with impaired vision have the anatomical potential to gain from participation in a clinical trial. We used supervised machine learning to predict foveal function from foveal structure in blue cone monochromacy (BCM), an X-linked congenital cone photoreceptor dysfunction secondary to mutations in the *OPN1LW/OPN1MW* gene cluster. BCM patients with either disease-associated large deletion or missense mutations were studied and results compared with those from subjects with other forms of IRD and various degrees of preserved central structure and function. A machine learning technique was used to associate foveal sensitivities and best-corrected visual acuities to foveal structure in IRD patients. Two random forest (RF) models trained on IRD data were applied to predict foveal function in BCM. A curve fitting method was also used and results compared with those of the RF models. The BCM and IRD patients had a comparable range of foveal structure. IRD patients had peak sensitivity at the fovea. Machine learning could successfully predict foveal sensitivity (FS) results from segmented or un-segmented optical coherence tomography (OCT) input. Application of machine learning predictions to BCM at the fovea showed differences between predicted and measured sensitivities, thereby defining treatment potential. The curve fitting method provided similar results. Given a measure of visual acuity (VA) and foveal outer nuclear layer thickness, the question of how many lines of acuity would represent the best efficacious result for each BCM patient could be answered. We propose that foveal vision improvement potential in BCM is predictable from retinal structure using machine learning and curve fitting approaches. This should allow estimates of maximal efficacy in patients being considered for clinical trials and also guide decisions about dosing.

## Introduction

Gene augmentation therapy clinical trials in inherited retinal degenerations (IRDs) have to date mainly delivered vector-gene product by the subretinal route (reviewed in Garafalo et al., 2019). A preferred surgical location for the induced retinal detachment has been the macular region. Among the many reasons for targeting the macula is the realization that, if proven safe, a therapy that increases or preserves central vision would be noticed and appreciated by patients. Also, monitoring of post-treatment efficacy could occur by common clinical methods such as visual acuity (VA) and central structural parameters using optical coherence tomography (OCT).

Many IRD patients with impaired foveal structure and vision could benefit from gene augmentation delivered to the retina with a less traumatic method than a surgically induced macular detachment which carries the potential risk of further reducing central vision. One potential option is an intravitreal delivery of vector-gene product. Among the caveats associated with intravitreal gene therapy are concerns about potency and inducing uveitis (Miller and Vandenberghe, 2018) and most experimental evidence in non-human primates is that mainly the photoreceptors in the fovea would be able to be targeted with the current viral vectors (Dalkara et al., 2013; Boye et al., 2016; Khabou et al., 2018; Byrne et al., 2020).

A number of IRDs would qualify as diseases in which an improvement in VA, despite foveal photoreceptor abnormalities, would be welcomed by affected patients, even if not accompanied by a gain in expanse of visual field (Garafalo et al., 2019). Among these IRDs is blue cone monochromacy (BCM), the X-linked congenital disorder with loss of red (L, long wavelength sensitive) and green (M, middle wavelength sensitive) cone photoreceptor function secondary to mutations in the *OPN1LW/OPN1MW* gene cluster on chromosome Xq28. There is evidence of retained L/M cones at and around the fovea in BCM, suggesting that this condition may be a candidate for intravitreal gene therapy (Cideciyan et al., 2013; Carroll et al., 2014; Scoles et al., 2016; Sumaroka et al., 2018; Garafalo et al., 2019). Missing to date, however, have been studies that would determine the level of efficacy that could be expected from such therapy, i.e., what is the degree of improvement in foveal function that could occur in the individual BCM patient.

Machine learning techniques have recently been used to predict treatment potential in two forms of Leber congenital amaurosis (LCA) caused by mutations in *CEP290* or *IQCB1* (*NPHP5*), that had little or no measurable vision but some measurable central retinal structure (Sumaroka et al., 2019). The present work uses these methods to try to predict from cross-sectional retinal structure images with OCT in BCM patients of different genotypes the best possible foveal visual outcomes in a clinical trial of intravitreally delivered vector-gene, acknowledging that it would only target foveal cones and not extracentral dysfunctional cones in these retinas.

## Materials and Methods

### Human Subjects

Two groups of patients were included: IRD patients (*n* = 26; ages 18–72 years; Supplementary Table S1) and BCM patients (*n* = 16; ages 7–52 years; Supplementary Table S2). IRD patients had retained visual acuities of at least 20/250 and measurable foveal cone function, determined as sensitivity to a 600-nm light stimulus on a white background light. All IRD patients had retained foveal outer nuclear layer thickness (range, 24–150 μm). We assumed that the cones in this cohort of IRD patients were functioning proportional to their remaining quantum catch and there was no additional de-sensitization beyond the partial loss of photoreceptors and shortening of outer segments (OSs) among the surviving cones (Sumaroka et al., 2019). Patients with cystoid macular edema or foveal atrophy were not included. Data from three normal subjects (N1–3, ages 22–32) were also included in this “training set” to cover the full range of foveal photoreceptor layer parameters. BCM patients included two eight-patient cohorts with different genotypes: those with large deletion mutations and those with the C203R missense mutation in a singular resident *OPN1LW* or *OPN1LW/MW* hybrid gene or in all genes of the *OPN1LW/OPN1MW* gene cluster (Sumaroka et al., 2018). All subjects underwent a complete eye examination as well as specialized tests of visual function and structure. Data from one eye were included for each patient. The research was approved by the institutional review board at the University of Pennsylvania. Previous genetic research testing in addition was approved by the institutional review board at the University of Tuebingen. All subjects were treated in accordance with the tenets of the Declaration of Helsinki; informed consent was obtained from adults, and assent with parental permission for all children.

### Foveal Structure: Optical Coherence Tomography (OCT), Data Extraction

Cross-sections along the horizontal meridian through the fovea were obtained with OCT (RTVue-100; Optovue Inc., Fremont, CA, United States). The principles of the method and our recording and analysis techniques have been published (e.g., Sumaroka et al., 2016, 2018). Three 15° wide B-scans composed of 4,091 A-scans or longitudinal reflectivity profiles (LRPs) were selected from each subject. Post-acquisition processing of OCT data was performed with custom programs (MATLAB Release, 2018, MathWork, Natick, MA, United States). All scans were aligned by straightening the major hyperreflective signal believed to originate near the interface between the basal aspect of the retinal pigment epithelium and Bruch’s membrane (RPE2/BrM). To increase the signal-to-noise ratio, lateral sampling density of the B-scan was reduced by averaging neighboring LRPs to get 512 A-scans per 15°. All scans were centered at the fovea; the foveola was identified manually as the maximum depression. Six retinal layers were identified and segmented with a computer-assisted algorithm: outer plexiform layer (OPL), external limiting membrane (ELM), inner segment (IS)/OS [also known as ellipsoid zone (EZ)] line, cone OS tips (COST; also known as phagosome zone; Cuenca et al., 2018, 2020), apical aspect of the RPE (RPE1), and RPE2/BrM (Supplementary Figure S1A). From this segmentation, ONL thickness, IS and COS length, and RPE thickness were extracted. Data were extracted at seven eccentricities centered at the fovea with 0.25° increments. In addition, the number of negative and positive peaks (extrema) on the gradient of the LRPs between the ELM and RPE2/BrM layers was automatically counted (Supplementary Figure S1B). The reflectivity values at each depth with respect to RPE2/BrM were extracted at the same eccentricity (Supplementary Figure S1C). The results of data extraction for ONL, IS, COS, and RPE are shown and the range of the values in the training set overlap with those of the BCM patient set (Supplementary Figure S1D).

### Foveal Function: Visual Sensitivity and Visual Acuity

Visual sensitivities were measured in the light-adapted state using 600-nm stimuli (size V, 1.7° diameter, 200 ms duration) on a standard 10 cd.m^–2^ white background (Humphrey Field Analyzer, HFA-750i analyzer, Zeiss-Humphrey, Dublin, CA, United States). The method has been described (for example, Charng et al., 2016; Matsui et al., 2016). Best-corrected VA was measured with the Early Treatment Diabetic Retinopathy Study (ETDRS) methodology.

### Data Analysis of the Training Set: Relationship of Structure and Function

We used two techniques of regression analysis to predict the possible outcome of treatment. The first technique, a supervised machine learning approach [random forest (RF)], was used to model the relationship between foveal function [foveal sensitivity (FS); VA] and retinal structure. Following our previous data analyses (Sumaroka et al., 2019), two groups of models based on the form of input variables were constructed. The model in each group was applied to predict FS and VA. Group 1 (Model I-FS and I-VA) used the input features derived from segmentation parameters: thicknesses of ONL, IS, COS, and RPE, and the number of distinct layers between ELM and RPE2/BrM. Additionally, the relationship between ONL and COS, and thickness of these layers and retinal eccentricity were accounted for by directly including interaction terms. For models in Group II (Model II-FS and II-VA), only reflectivity values were used as input features. Performance of each model was evaluated by leave-one-out, 29-fold cross-validation. The predicted value was defined as average of prediction at each eccentricity in all three scans based on the remaining subjects in the training set.

The second technique was based on curve fitting using the established relationship between photopigment (and outer retinal structure) and visual-retinal thresholds, measured by psychophysics or electrophysiology (Ripps et al., 1978; Machida et al., 2000; Jacobson et al., 2005, 2014). To implement fitting, we used the relationship between retinal structural data and functional data derived from our training set (Supplementary Figure S1E). In this technique, the predicted result was defined as the average of three predictions using foveal ONL (0-eccentricity).

Final RF models were trained using data from all 29 subjects (26 IRD and three normals). Root-mean-square error (RMSE) was used as an indicator of model performance for all methods. The difference between the measured target variable (sensitivity) and the predicted value was calculated first. Difference across all patients was squared, averaged, and square root extracted. Range of predictions were estimated as ± 1.96^∗^RMSE.

## Results

### Machine Learning to Predict Foveal Function From Foveal Structure in IRDs

**FIGURE 1:**
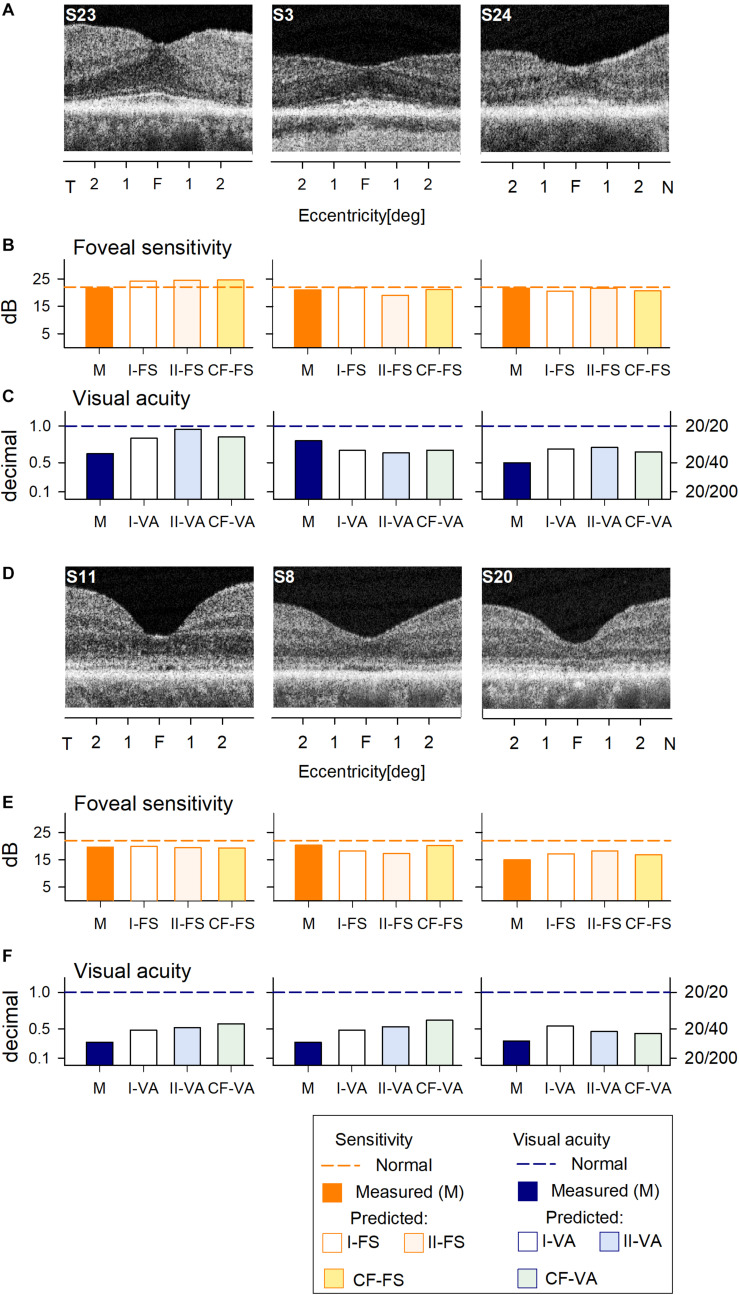
Prediction of foveal sensitivity (FS) and visual acuity (VA) in the IRD training set group. **(A)** Foveal region OCT scans from 3 IRD subjects (S23, S3, S24) representing different ONL thicknesses. **(B)** Comparison of measured FS (M, orange bar) and predicted results by Model I-FS (white bar) and Model II-FS (light orange bar). Yellow bar is FS value calculated by formula extracted from curve fitting (CF-FS). **(C)** Comparison of measured VA (dark blue bar) and predicted by Model I-VA (white bar) and Model II-VA (light blue bar). Light green bar is VA calculated by a formula extracted from curve fitting (CF-VA). **(D–F)** are the same as **(A–C)** but the scans are from 3 IRD subjects (S11, S8, S20) who have visible defects of IS/OS similar to those encountered in some BCM patients. Dashed lines in **(B,C,E,F)** represent lower boundary of normal range.

We first asked whether the machine learning algorithm can be trained to predict reliably the foveal cone sensitivity based on co-localized foveal retinal structure in a cohort of patients with different forms of IRD. An RF supervised learning algorithm was used with the foveal structure parameters as input and light-adapted 600 nm sensitivity as output (Figure 1). Measured and predicted FSs were compared for all IRD patients. Data from six IRD patients illustrate a range of thicknesses of foveal ONL (Figures 1A,D) along with the corresponding measured FS and VA in comparison with predictions by two models for each parameter (I-FS, II-FS and I-VA, II-VA, respectively) (Figures 1B,C,E,F). S23 and S3 exemplify milder central retinal disease with normal or near-normal FS and retained IS/OS structure, whereas S24 has more severe disease with greater foveal abnormalities in function and structure (Figure 1A). S11, S8, and S20 illustrate abnormal foveae with visible defects of IS/OS (Figure 1D) similar to those in some BCM patients. Measured FSs in these patients (especially S20) are further reduced and there is corresponding loss of VA.

Measured and predicted FS were compared for the six IRD patients representing a spectrum of disease severities. The predictions of the models appear to approximate well the measured FS and VA values (Figures 1B,C,E,F).

**FIGURE 2:**
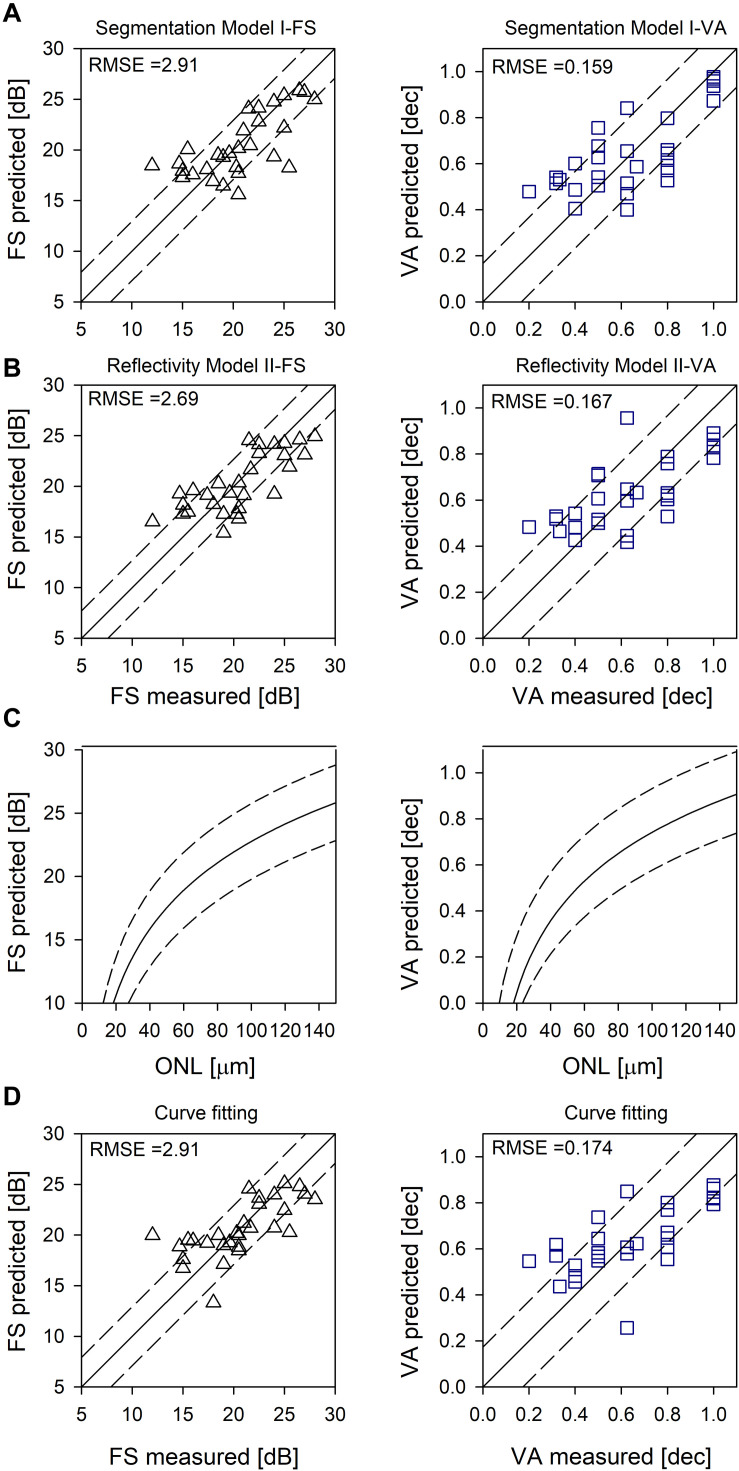
Evaluation of the models. **(A)** Random forest segmentation Model I-FS and I-VA showing predicted versus measured values. **(B)** Random forest Model II-FS and II-VA showing predicted versus measured values. Equality line (y = x) is superimposed (solid line); RMSE are shown as dashed lines **(A,B)**. **(C)** Logarithmic curve [y = y0 + a*log(x)] fitted to FS versus ONL, and VA versus ONL (solid lines). **(D)** Prediction based on curve fitting. Equality line (y = x) is superimposed (solid line). RMSE are shown as dashed lines **(C,D)**.

Across all IRD patients, differences between measured and predicted sensitivities were used to calculate RMSE for each model (Figures 2A,B).

### Curve Fitting to Predict Foveal Function From Foveal Structure in IRDs

Using non-linear regression analysis, we fit a logarithmic curve that assumes the cones were functioning proportional to their remaining quantum catch (following the experimental results in Ripps et al., 1978; Machida et al., 2000; Jacobson et al., 2005, 2014). The function used was y = y0 + a^∗^log(x), where y was either FS in dB or VA in decimal and x was foveal ONL thickness in microns. Coefficients for FS were y0 = −12.01, (*p* < 0.01) a = 17.38 (*p* < 0.01) and for VA were y0 = −1.07, (*p* < 0.01) a = 0.91 (*p* < 0.01) (Figure 2C, solid line). Examples of the predicted foveal function by calculating FS (Figures 1B,E) and VA (Figures 1C,F) using only foveal ONL as input are shown. Calculated versus measured FS and VA are plotted across all IRD patients (Figure 2D) and an equality line is superimposed (solid line); RMSE was estimated from the difference between measured and calculated FS and VA (dashed lines, Figures 2C,D).

### Predicted and Measured Sensitivities in BCM

Predictions of FS and VA from foveal structure were then tested in patients with BCM. The BCM patients had either disease-associated large deletion mutations or the C203R mutation (Supplementary Tables S1, S2). The two categories of genotypes were recently found to have different phenotypes in terms of persistence of foveal structure; patients with large deletions show on average more severe losses of central structure at earlier ages (Sumaroka et al., 2018). The cohort of eight patients with large deletion mutations tended to be younger in age than those with the C203R mutation.

**FIGURE 3:**
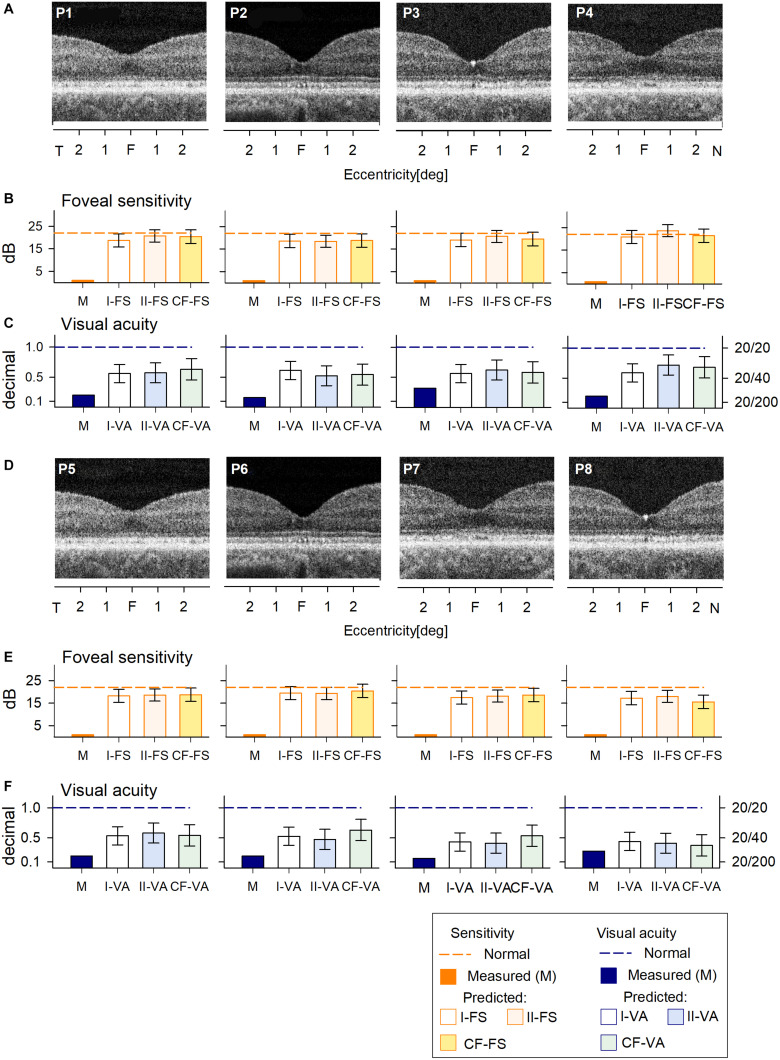
Prediction of FS and VA of BCM patients with large deletion mutations. **(A)** Foveal region OCT scans from four BCM patients (ages 7–13 years). **(B)** Non-detectable sensitivity at foveal location (orange bar) and predicted results by Model I-FS (white bar) and Model II-FS (light orange bar) as in Figure 1. Yellow bar is sensitivity value calculated by formula extracted from curve fitting (CF-FS). **(C)** Comparison of measured VA (dark blue bar) and predicted results by Model I-VA (white bar) and Model II-VA (light blue bar). Light green bar is visual acuity calculated by formula extracted from curve fitting (CF-VA). **(D–F)** are the same as **(A–C)** but BCM patients are older (ages 18–35 years); dashed lines in **(B,C,E,F)** represent lower boundary of normal range. Error bars represent RSME for corresponding model.

All of the patients with large deletions had no detectable FS at the foveal location. Predicted FS results by the two RF models and the curve fitting were similar and indicated the potential for at least 1.5 log unit FS increases (Figures 3B,E). As expected, VA was reduced in all patients; the predicted results by both RF models and curve fitting indicated a comparable level of improvement potential (Figures 3C,F). There was no difference between results that explicitly took into consideration cone OS (COS) length (Model I-VA) and the other two approaches (II-VA, CF).

**FIGURE 4:**
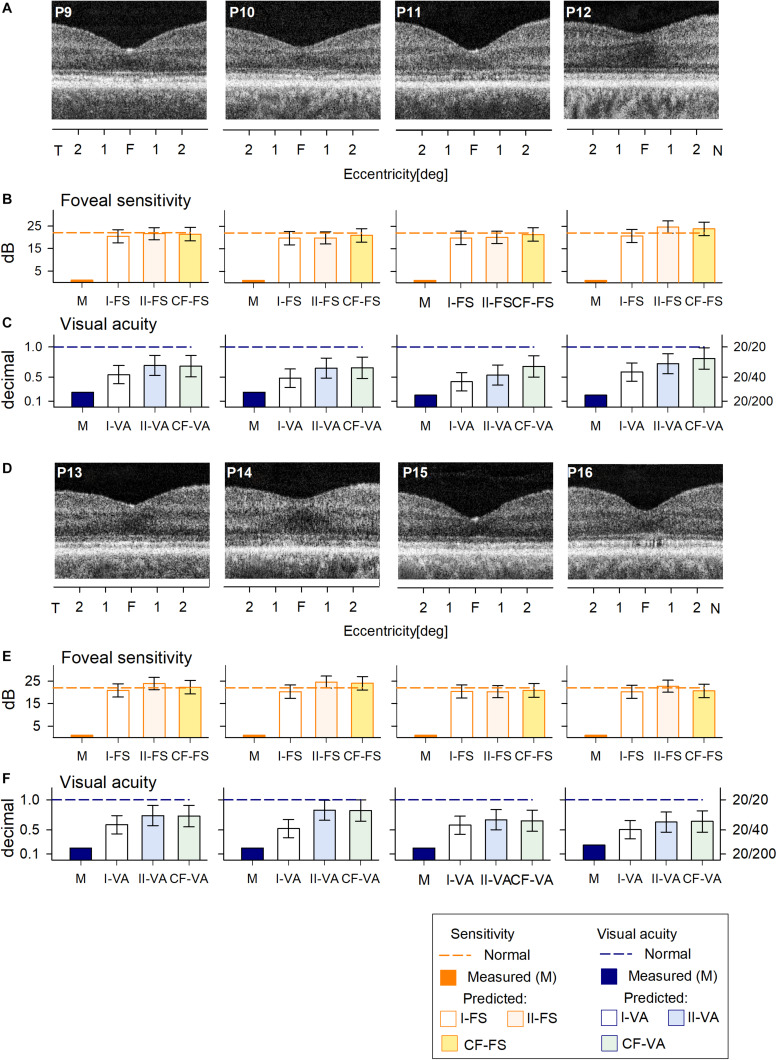
Prediction of FS and VA of BCM patients with the C203R mutation. **(A)** Foveal region OCT scans from four BCM patients (ages 13–35 years). **(B)** Non-detectable sensitivity at foveal location (orange bar) and predicted results by Model I-FS (white bar), Model II-FS (light orange bar), and CF-FS (yellow bar) as in Figures 1, 3. **(C)** Comparison of measured VA (dark blue bar) and predicted and predicted by Model I-VA (white bar), Model II-VA (light blue bar), and CF-FS (light green bar). **(D–F)** are the same as **(A–C)** but BCM patients are older (ages 35–52 years); dashed lines (in **B**,**C**,**E**,**F**) represent lower boundary of normal range. Error bars represent RSME for corresponding model.

The cohort of C203R BCM patients also had no detectable FS (Figures 4B,E) and reduced VA (Figures 4C,F). RF models and curve fitting predicted potential increase in FS but some patients (P12, P13, P14) showed slightly greater increases when COS length was not considered (I-FS). This suggests that these BCM foveae with greater ONL thickness but shorter COS than expected had less potential for improvement. This was even more evident in the VA data; Model I-VA tended to predict less improvement than the other two approaches (II-VA, CF).

**FIGURE 5:**
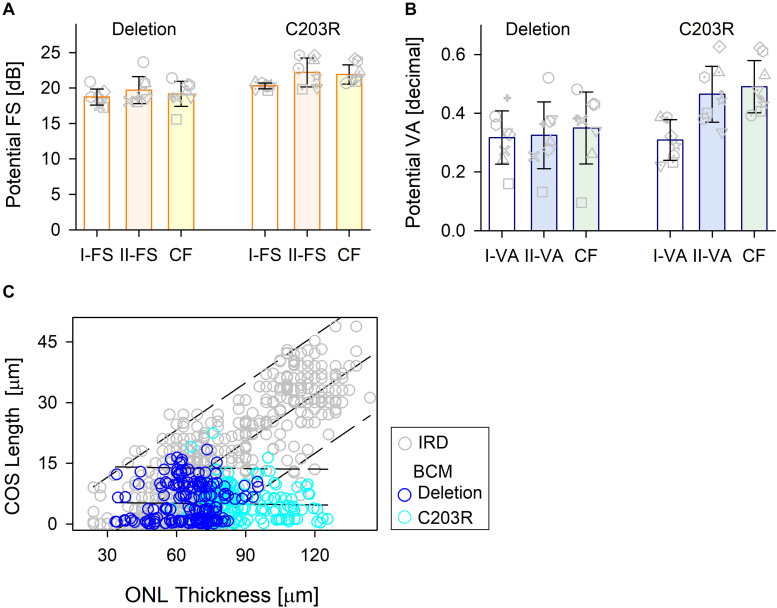
Comparison of the treatment potential (difference between prediction and measured values) for the three methods in the two BCM genotypes. **(A)** Treatment potential for FS in deletion mutations and C203R mutation patients. Model 1, white bar; Model II, light orange bar; CF, light yellow bar. **(B)** Treatment potential for VA in the two groups. Model 1, white bar; Model II, light blue bar; CF, light green bar. Individual subjects, gray unfilled symbols; error bars, standard deviation. **(C)** Relation between ONL thickness and COS length for IRD patients used in the training set (gray circles) and those for BCM patients (deletion, dark blue circle; C203R, cyan circle).

Comparison of the treatment potential (difference between prediction and measured values) based on data from the three methods is shown for the two BCM genotype groups (Figure 5). For the patients with large deletions, all three approaches were, on average, the same. For C203R mutation patients, there was a statistically significant difference between approaches (Kruskal–Wallis one-way ANOVA on Ranks, *P* = 0.021); Model I-FS was on average less than II-FS and CF (Student–Newman–Keuls method *P* < 0.05) (Figure 5A). A similar effect was observed with VA predictions with little or no difference for patients with large deletions but considerable difference in the C203R patients (one-way ANOVA, *P* < 0.001, Student–Newman–Keuls method *P* < 0.05 (Figure 5B). Plotting ONL versus COS thicknesses for training and BCM sets indicates there is a difference. For the training set, there was strong correlation between COS length and ONL thickness (*r* = 0.814, *P* < 0.001), as has been observed for rod structure by histopathology (Machida et al., 2000). For BCM, there is no correlation of COS and ONL; COS thickness is reduced and independent of genotype. The greater COS thickness for the C203R cohort for the same ONL thickness as in the training set subjects would explain the difference in the prediction between Model I versus Model II as well as CF for the two genotypes (Figure 5C).

## Discussion

Current understanding of the X-linked human disease BCM was preceded by a lengthy journey of scientific discovery that included the basics of photoreceptor anatomy, biochemistry, physiology, and molecular genetics (for example, Young, 1802; von Helmholtz, 1866; Osterberg, 1935; Nathans et al., 1986a, b, 1989, 1993; Curcio et al., 1990; Saari, 2000; Neitz and Neitz, 2011; Palczewski, 2012). Recent progress in delivery of genes to the retina has prompted discussion of which disease entities would be promising targets for gene therapies (Garafalo et al., 2019). One of the first questions to ask about BCM as a possible candidate for gene augmentation therapy was whether there were sufficient L/M cone photoreceptors present in the retina of these patients, considering the disease manifests as a congenital visual disorder and there is a macular degenerative component at later stages (Cideciyan et al., 2013). For BCM due to large deletion or C203R mutations included in the current work, the hypotheses have evolved over time. Earlier work based on adaptive optics (AO) images of waveguiding cones in C203R patients suggested that residual cones were all S cones and there were no L/M cones to be treated (Carroll et al., 2012). In contrast, evaluation of the spatial density of dark (non-waveguiding) cones together with OCT measurements in deletion patients was consistent with partially retained L/M cones (Cideciyan et al., 2013). Similarly retained L/M cones were later found in C203R patients with the use of split-detector AO imaging of their ISs (Carroll et al., 2014; Scoles et al., 2016; Patterson et al., 2020). More recently, the question of genetic heterogeneity and possible phenotypic differences within BCM was addressed with studies of cohorts of patients with large deletions versus patients with missense mutation, specifically C203R. An unexpected observation was that foveal cone structure was more persistent in the cohort of patients with the C203R mutation and the difference in natural history of disease progression could be as much as two to three decades (Sumaroka et al., 2018). Examination of further C203R patients in the sixth and seventh decades of life confirmed the original findings (Sumaroka, unpublished observation). Despite these structural differences in foveal photoreceptor integrity between genotypes at different patient ages, there were no significant differences in central visual function, such as VA (Sumaroka et al., 2018).

Is there progress toward therapy for the visual dysfunction in BCM? Recent murine proof-of-concept studies lend support to the concept that a gene augmentation approach to BCM may be feasible (Zhang et al., 2017; Deng et al., 2018, 2019). What is the expectation for therapeutic efficacy in a patient with BCM? We determined that there are sufficient cone photoreceptors to warrant foveal gene therapy in BCM (Cideciyan et al., 2013) but not until the current study were we able to ask about the potential difference to central visual function that efficacious therapy could make. The opportunity to ask such a question is presented by the considerable progress in technology of non-invasive imaging, and understanding of the anatomical basis of the cross-sectional retinal images with OCT (for example, Huang et al., 1998; Podoleanu and Rosen, 2008; Spaide and Curcio, 2011; Muller et al., 2019). Our first such attempt at predicting vision from retinal structure was done in two forms of LCA caused by *CEP290* and *IQCB1* (*NPHP5*) mutations that retained foveal and extrafoveal cone islands but no evidence of rods. The supervised machine learning approach used data from patients with retinitis pigmentosa with only cone-mediated macular function remaining. The results in these two forms of severe genetic retinal blindness allowed for prediction of maximal efficacy (Sumaroka et al., 2019).

The present work asked a similar question about prediction of vision from OCT structure but the differences between BCM and the previously studied forms of LCA required changes in strategy. Ideally, any treatment of BCM would target all the L/M cones throughout the retina but that will have to await future advances in gene (or other therapeutic) delivery. There is no evidence that L/M cones in human BCM are structurally intact outside of the fovea; the retinal region feasible to target would be the BCM fovea. BCM gene therapy would preferably be via an intravitreal delivery, and intravitreal gene delivery has been mainly limited to foveal transduction (Dalkara et al., 2013). Therefore, in BCM only the fovea and foveal cone photoreceptor structure would need to be quantified; surrounding extrafoveal retina where rod:cone ratios increase rapidly would not be targeted and, unlike the *CEP290* and *IQCB1* (*NPHP5*) forms of LCA previously analyzed, BCM has evidence of normal rod function and structure (Curcio et al., 1990; Cideciyan et al., 2013). Limiting the targeted retinal area to the fovea is useful in the design of a clinical trial because the functional predictions would best include the time-honored measure of central spatial vision (i.e., VA) as well as a measurement of visual sensitivity at fixation that is dramatically reduced in BCM (e.g., longer wavelength stimuli in the light-adapted state).

Two techniques of regression analysis were used to predict the possible outcome of therapy and the results were compared. A supervised machine leaning approach (RF) was taken to model the relationship between retinal structure and both FS and VA. This followed our previous data analyses: two groups of models based on the form of the input variables were constructed (Sumaroka et al., 2018). The second technique used CF to known foveal function and structural data (ONL) and this was based on an established model assuming photoreceptor function proportional to remaining quantum catch (Ripps et al., 1978; Kemp et al., 1988; Machida et al., 2000; Jacobson et al., 2005, 2014; Rangaswamy et al., 2010); parameters of a mathematical equation describing their relationship were extracted. RF models and CF produced comparable predictions of foveal efficacy in the BCM patients.

How would a BCM clinical trial use the data and analyses from the current study? In the present era of novel therapies, we enter early phase trials with the primary goal of evaluating safety; there is usually a qualitative prediction that there may be some efficacy signal at the doses initially used. A pattern has been that if VA improves (to some degree) compared to baseline, success is announced, and the trial may actually move forward to later phases, given regulatory approval of course. For BCM [and *CEP290*-LCA and *IQCB* (*NPHP5*)-LCA; Sumaroka et al., 2019], we can now advance to quantitative prediction of efficacy outcome. Once a patient has a clinical and molecular diagnosis of BCM, relevant parameters of function and structure would be quantified. The most commonly available measure of central visual function in the clinic is best-corrected VA, and retinal structure would be measured using OCT scans through the fovea. The presence of foveal ONL (by observation of the scan) would likely fulfill an entry criterion for enrollment. Yet, there is a need for quantitation of foveal ONL, and OCT machine algorithms are up to this task. VA and ONL at baseline allow us to estimate efficacy using predicted VA (VA_p_) calculated from the following formula: VA_p_ = −1.07 + 0.91^∗^log(ONL). From the result, a patient’s predicted efficacy from therapy would be determined as VA_p_–VA. For example, P13 (C203R mutation, in his fourth decade of life) with a VA of 20/100 (decimal 0.2) and foveal ONL thickness of 90 μm would be predicted to show on average an improvement in VA to 20/25 (decimal 0.71 ± 0.34), equivalent to a six-line positive change (range four to seven lines) on an ETDRS chart. A further example is P5 (deletion mutation, in his second decade of life) with the same VA of 20/100 (decimal 0.2) as P13 but foveal thickness of 59 μm would be predicted to show an improvement in VA to 20/40 (decimal 0.54 ± 0.34), equivalent to a four-line positive change on an ETDRS chart (range zero to six lines). Treatment potential is estimated based on the retinal structure retained by each patient at the time of the intervention. If treatment changed the retinal structure, such as leading to COS lengthening, foveal treatment potential would also be expected to increase accordingly. Results in a recent clinical trial in *CEP290*-LCA suggested that such positive structural changes in the outer retina are possible (Cideciyan et al., 2019).

Other progress using machine learning to predict measures of visual function or retinal structure from various inputs is published and the topic is now being reviewed frequently (for example, Schmidt-Erfurth et al., 2018; Arcadu et al., 2019; Kihara et al., 2019; Wen et al., 2019; Della Volpe-Waizel et al., 2020). For the field of IRDs, next steps would be to research how artificial intelligence-based modeling could be used to analyze conditions with residual rod-mediated vision and structure. Ongoing and future gene-based trials that involve rod photoreceptors would benefit from such predictions.

## Data Availability Statement

The raw data supporting the conclusions of this article will be made available by the authors, without undue reservation.

## Ethics Statement

The studies involving human participants were reviewed and approved by the University of Pennsylvania, Institutional Review Board. Written informed consent to participate in this study was provided by the participants’ legal guardian/next of kin.

## Author Contributions

AS, AC, SK, BW, and SJ contributed to the experimental design and performed experiments, data analysis, and manuscript writing. RS and VW performed data analysis and editing. All authors contributed to the article and approved the submitted version.

## Conflict of Interest

The authors declare that the research was conducted in the absence of any commercial or financial relationships that could be construed as a potential conflict of interest.
