# Supplementary figures and images for: Foveal Therapy in Blue Cone Monochromacy: Predictions of Visual Potential From Artificial Intelligence
Source: Front Neurosci. 2020 Aug 3;14:800. doi: 10.3389/fnins.2020.00800 (PMC7416698; doi:10.3389/fnins.2020.00800)

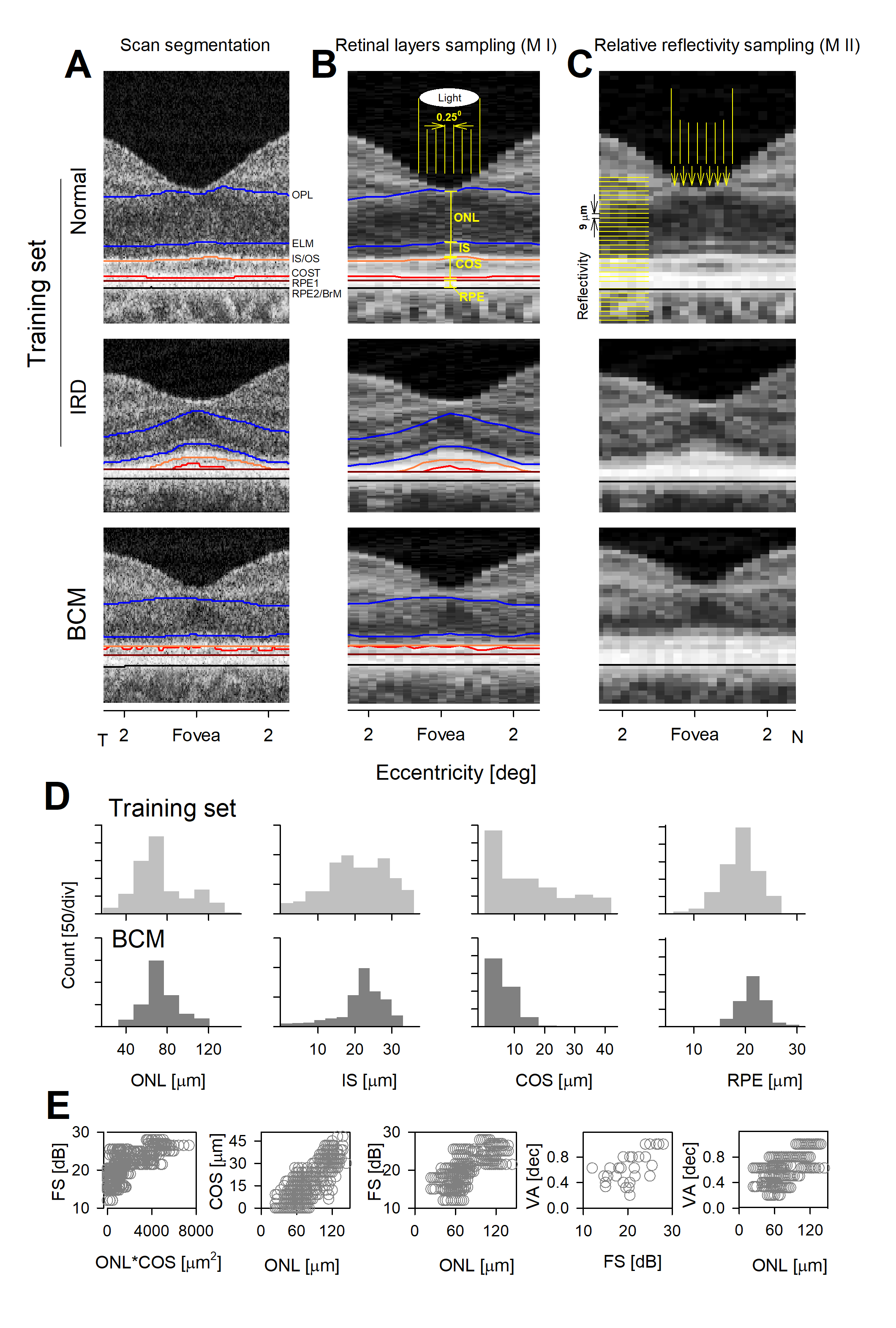

Supplement: Supplementary file 1 [file Image_1.TIF]
