# Supplementary material for: Foveal Therapy in Blue Cone Monochromacy: Predictions of Visual Potential From Artificial Intelligence
Source: Front Neurosci. 2020 Aug 3;14:800. doi: 10.3389/fnins.2020.00800 (PMC7416698; doi:10.3389/fnins.2020.00800)
Supplement: Supplementary file 2 [file Table_1.DOC]

**ORIGINAL RESEARCH ARTICLE**

**Frontiers in Neuroscience: Retinal Degeneration and Therapy Approaches**

**Foveal Therapy in Blue Cone Monochromacy: Predictions of Visual Potential from Artificial Intelligence**

***Alexander Sumaroka, Artur V. Cideciyan, Rebecca Sheplock, Vivian Wu, Susanne Kohl, Bernd Wissinger, and Samuel G. Jacobson***

**SUPPLEMENTARY MATERIALS**

**SUPPLEMENTARY FIGURE S1.** Structural data extraction in representative normal, IRD subject and BCM patient. **(A)** Segmentation of six layers in scans of the foveal region. **(B)** White ellipse (representing light stimulus, 1.7^o^ diameter) above scan and seven samples (at 0.25^o^ intervals) under the ellipse showing where thickness data for ONL (outer nuclear layer), IS (inner segment), COS (cone outer segment) and RPE retinal pigment epithelium) were collected. **(C)** Scheme for collecting reflectivity data over this same region. **(D)** Distribution of the parameters in training set (light gray) and BCM patient set (dark gray). **(E)** Observed relationships of structural and functional data. Left to right panels: FS versus ONL*COS; ONL versus COS; FS versus ONL; VA versus FS; and VA versus ONL.

| **SUPPLEMENTARY TABLE S1.** Clinical characteristics of subjects used in training random forest algorithm | | | |
| --- | --- | --- | --- |
| Subjects | Age at visit [range] | Best-corrected Visual Acuity* | Clinical Diagnosis‡ |
| S1 | 16 - 20 | 20/25 | XLRP |
| S2 | 16 - 20 | 20/40 | XLRP |
| S3 | 16 - 20 | 20/25 | XLRP |
| S4 | 16 - 20 | 20/32 | XLRP |
| S5 | 21 - 25 | 20/32 | BBS |
| S6 | 21 - 25 | 20/25 | XLRP |
| S7 | 21 - 25 | 20/50 | XLRP |
| S8 | 21 - 25 | 20/63 | CRD |
| S9 | 26 - 30 | 20/32 | XLRP |
| S10 | 26 - 30 | 20/25 | XLRP |
| S11 | 26 - 30 | 20/63 | Maculopathy |
| S12 | 26 - 30 | 20/100 | RP |
| S13 | 31- 35 | 20/40 | XLRP |
| S14 | 31- 35 | 20/30 | XLRP |
| S15 | 36 - 40 | 20/40 | CRD |
| S16 | 41 - 45 | 20/32 | XLRP |
| S17 | 41 - 45 | 20/50 | USH |
| S18 | 41 - 45 | 20/25 | XLRP |
| S19 | 46 - 50 | 20/63 | RP |
| S20 | 46 - 50 | 20/60 | CRD |
| S21 | 51 - 55 | 20/20 | RP |
| S22 | 56 - 60 | 20/25 | USH |
| S23 | 56 - 60 | 20/32 | RP |
| S24 | 56 - 60 | 20/40 | RP |
| S25 | 61 - 65 | 20/40 | USH |
| S26 | 71 - 75 | 20/20 | RP |
| N1 | 18 - 25 | 20/20 | Normal |
| N2 | 31 - 35 | 20/20 | Normal |
| N3 | 31 - 35 | 20/20 | Normal |
| * Best-corrected visual acuity for eye with OCT used in training given in Snellen  ‡ Abbreviations are given as follows: RP, retinitis pigmentosa; XLRP, X-linked retinitis pigmentosa; USH, Usher syndrome; BBS, Bardet-Biedl syndrome; CRD, cone-rod dystrophy | | | |

| **SUPPLEMENTARY TABLE S2.** Clinical characteristics of BCM patients | | | | |
| --- | --- | --- | --- | --- |
| Mutation type: *OPN1LW/OPN1MW* gene cluster | Patient | Age at visit [range] | Best Corrected  Visual Acuity* |  |
| Large deletion | P1 | 6 - 10 | 20/100 |  |
|  | P2 | 11 - 15 | 20/125 |  |
|  | P3 | 11 - 15 | 20/100 |  |
|  | P4 | 11 - 15 | 20/63 |  |
|  | P5 | 16 - 20 | 20/100 |  |
|  | P6 | 16 - 20 | 20/100 |  |
|  | P7 | 26 - 30 | 20/125 |  |
|  | P8 | 31 - 35 | 20/63 |  |
| C203R missense | P9 | 11 - 15 | 20/80 |  |
|  | P10 | 16 - 20 | 20/80 |  |
|  | P11 | 21 - 25 | 20/100 |  |
|  | P12 | 31 - 35 | 20/100 |  |
|  | P13 | 31 - 35 | 20/100 |  |
|  | P14 | 36 - 40 | 20/100 |  |
|  | P15 | 41 - 45 | 20100 |  |
|  | P16 | 51 - 55 | 20/80 |  |
| * Visual acuity for eye with OCT used in analysis given in Snellen | | | | |
